# The effect of alcohol on the differential expression of cluster of differentiation 14 gene, associated pathways, and genetic network

**DOI:** 10.1371/journal.pone.0178689

**Published:** 2017-06-02

**Authors:** Diana X. Zhou, Yinghong Zhao, Jessica A. Baker, Qingqing Gu, Kristin M. Hamre, Junming Yue, Byron C. Jones, Melloni N. Cook, Lu Lu

**Affiliations:** 1 Department of Genetics, Genomics and Informatics, University of Tennessee Health Science Center, Memphis, Tennessee, United States of America; 2 Department of Neurology, Affiliated Hospital of Nantong University, Nantong, China; 3 Department of Anatomy and Neurobiology, University of Tennessee Health Science Center, Memphis, Tennessee, United States of America; 4 Department of Cardiology, Affiliated Hospital of Nantong University, Nantong, China; 5 Department of Pathology, University of Tennessee Health Science Center, Memphis, Tennessee, United States of America; 6 Department of Psychology, University of Memphis, Memphis, Tennessee, United States of America; Oregon Health and Science University, UNITED STATES

## Abstract

Alcohol consumption affects human health in part by compromising the immune system. In this study, we examined the expression of the *Cd14* (cluster of differentiation 14) gene, which is involved in the immune system through a proinflammatory cascade. Expression was evaluated in BXD mice treated with saline or acute 1.8 g/kg i.p. ethanol (12.5% v/v). Hippocampal gene expression data were generated to examine differential expression and to perform systems genetics analyses. The *Cd14* gene expression showed significant changes among the BXD strains after ethanol treatment, and eQTL mapping revealed that *Cd14* is a *cis*-regulated gene. We also identified eighteen ethanol-related phenotypes correlated with *Cd14* expression related to either ethanol responses or ethanol consumption. Pathway analysis was performed to identify possible biological pathways involved in the response to ethanol and *Cd14*. We also constructed a genetic network for *Cd14* using the top 20 correlated genes and present several genes possibly involved in *Cd14* and ethanol responses based on differential gene expression. In conclusion, we found *Cd14*, along with several other genes and pathways, to be involved in ethanol responses in the hippocampus, such as increased susceptibility to lipopolysaccharides and neuroinflammation.

## Introduction

Numerous studies have shown that the consumption of alcohol affects human health. One of the many ways that alcohol can exert its effects is by altering and subsequently lowering immune function [1]. Alcohol affects immune function through its effects on innate immune function/responses (i.e. monocytes, macrophages, natural killer cells and neutrophils among others) and adaptive immune function/responses (i.e. CD4 and CD8 T cells, Th1, 2, and 17 cells, and B cells among others as reviewed in [2,3]). These effects are dependent upon the pattern of alcohol exposure, which diversely influences the immune system and function. Inflammation, one of the body’s first defenses against pathogens, is both attenuated by moderate alcohol intake and increased by heavy alcohol consumption, creating a U-shaped relationship between alcohol and inflammation [4]. Alcoholism, such as chronic or heavy alcohol consumption, has been described as an inflammatory condition which can affect inflammation of the liver, intestines, lungs, and brain [5]. Alcohol consumption affects inflammatory processes through its effect to suppress the production of inflammatory mediators. It also compromises the integrity of the lining of the gastrointestinal tract and lungs, causes defects in granulocytes, inhibits their function, and impairs the antigen-presenting factor of dendritic cells [1]. While most immunosuppression associated with alcohol consumption does not cause significant effects, it does leave the body more susceptible to secondary immune insults. For example, lung inflammation and interleukin-18-mediated neutrophil infiltration after burn injury in rats are increased by acute alcohol intoxication [6].

*Cd14* (cluster of differentiation 14) is a gene vital in the immune system’s inflammatory cascade. It is a cell membrane-bound glycoprotein that is largely expressed in monocytes, macrophages, and microglial cells. This protein is also a co-receptor of TLR4 (one of several toll-like receptors which are integral in innate immune responses), particularly in the detection and binding of lipopolysaccharides, or LPS, which are found on the membranes of Gram-negative bacteria. Many studies have shown strong evidence linking *Cd14* to alcohol. Deletion of *Cd14*, along with other neuroimmune genes such as *Il6*, decreased alcohol consumption in null mutant mice, which suggest that neuroimmune signaling plays a role in alcohol-related behaviors [7]. Alcohol also increases gut permeability to LPS [8], which is then detected by CD14 and activates the cell, initiating a proinflammatory cascade [9] and stimulating the release of proinflammatory cytokines, including IL-6, IL-1, and TNF-α. In addition to affecting the innate immune system through inflammatory changes, alterations in CD14 function by alcohol exposure are also associated with the neuroinflammation and brain damage associated with alcohol misuse [10]. Multiple studies have shown that *Cd14*’s function is altered by ethanol consumption. However, the mechanisms through which *Cd14* regulates ethanol response and interacts with other genes are still unclear.

Either acute or chronic ethanol exposure is considered as a stressor. The sensitivity of the hippocampus to various stressors is well documented [11–14]. At both immunological and neurological levels, alcohol has deleterious effects on the hippocampus. Studies have shown that chronic alcohol use is associated with morphological changes in the hippocampus, including decreased hippocampal volume [15, 16]. Ethanol exposure also changes CD14 and TLR4 expression; while ethanol decreases neurogenesis in the hippocampus, neurogenesis is increased in TLR4 knockout mice [17]. The inflammatory cascade in the brain induced by alcohol consumption has also been found to increase the duration and magnitude of proinflammatory cytokines and microglial activation, thus increasing susceptibility to chronic illnesses [9]. One such chronic illness linked to *Cd14* and neuroinflammation is Alzheimer’s disease, which is heavily implicated with the hippocampus and can be detected in the hippocampal formation at an early stage before further symptoms arise [18, 19]. Perhaps more importantly, our previous studies have shown that gene expression in the hippocampus is particularly sensitive to the effects of acute ethanol (1.8 g/kg) [20, 21]. Others have also shown that acute ethanol (2.0 g/kg) produces brain region-specific changes in gene expression, including in the hippocampus [22]. Thus, the hippocampus is an excellent target for examining how *Cd14* potentially regulates ethanol responses. Further, the examination of acute ethanol treatment may allow for the identification of mechanisms, including gene networks that are also important in the cascade of events that lead to addiction and other chronic illnesses.

Recombinant inbred (RI) strains of mice are a very useful resource to identify the genetic basis of phenotypes, including the regulation of gene expression and genetic networks. The largest panel of these strains–the BXD family–consists of the inbred progeny of a cross between C57BL/6J (B6) and DBA2/J (D2) mice. Each BXD line is a discrete population with fixed genotypes at each locus and the parental B6 and D2 alleles segregating among the strains. Because individuals within each RI line are isogenic, the genotype of each line can be used to develop a map of complex traits ranging from DNA variation to phenotype. The BXD lines have been used extensively in genetic and genomic studies of many phenotypes and diseases, including ethanol consumption and anxiety phenotypes [23]. Importantly, others have shown that genetic factors contribute to variation in both hippocampal structure and function in this RI panel [24]. The BXD RI panel has also been useful in identifying molecular and genetic mechanisms influencing ethanol-induced changes in the brain [25].

The purpose of this study was to combine the power of RI strains of mice and a systems genetics approach to explore the role of *Cd14* in ethanol responses using two sets of existing hippocampal gene expression data that we generated. In this study we sought to identify expression quantitative trait loci (eQTL) for *Cd14*, analyze potential pathways through which *Cd14* interacts with ethanol, and construct a genetic network that plays a part in ethanol responses.

## Materials and methods

### Animals

Two groups of BXD recombinant inbred (RI) strains of mice were used in this study. The first set consisted of 67 BXD RI strains, which were used to generate hippocampal gene expression data for system genetics analysis. The second set consisted of 26 BXD RI strains that were used for saline or alcohol treatment.

All mice were group housed (2–5 mice in same sex cages) and maintained on a 12:12 light/dark cycle at University of Tennessee Health and Science Center (UTHSC) for the first set of 67 BXD RI mice and at University of Memphis for the second set of 26 BXD RI mice. When mice were sacrificed for tissue harvest, they were anesthetized using Avertin (1.25% 2,2,2- tribromoethanol and 0.8% tert-pentyl alcohol in water; 0.8–1.0 ml, i.p.) and killed via cervical dislocation. All animal work and experimental protocols for this specific study were approved by the UTHSC Institutional Animal Care and Use Committee (IACUC) and the Univeristy of Memphis Institutional Animal Care and Use Committee (IACUC) following NIH guidelines.

### Ethanol treatment

The B6, D2, and 26 BXD strains of mice (8–10 mice per strain including both males and females) were divided into two groups: 1) saline group, treated with iusovolumetric saline via IP injection, or 2) ethanol group, treated with an IP injection of 1.8 g/kg i.p. ethanol (12.5% v/v). These conditions allowed us to test for differences in gene expression both in the presence and absence of alcohol. Four hours after treatment, these mice were sacrificed for tissue harvest.

### Tissue harvest

The 67 untreated BXD RI strains used to generate hippocampal expression data and the ethanol or saline treated animals (26 BXD RI strains) described above, were killed and harvested for their tissue according to previous methods [26]. Briefly, mice were anesthetized using Avertin (1.25% 2,2,2- tribromoethanol and 0.8% tert-pentyl alcohol in water; 0.8–1.0 ml, i.p.) and killed via cervical dislocation at 2–6 months of age. The brain was extracted and dissected to obtain the hippocampus. The cortex above the hippocampus and dentate gyrus was removed along the septotemporal axis. The exposed hippocampus and dentate gyrus were then taken out of the hemisphere in a ventral-to-dorsal direction. The left and right hippocampi were pooled and stored in RNAlater overnight at 4°C, then kept at 80°C until RNA extraction.

### RNA extraction and microarrays

RNA was extracted from the hippocampus using RNA STAT-60 (protocols can be found at Tel-Test, www.tel-test.com) as per the manufacturer’s instructions. A spectrophotometer (Nanodrop Technologies, found at http://www.nanodrop.com) was used to measure RNA concentration and purity, and the Agilent 2100 Bioanalyzer was used to evaluate RNA integrity. The RNA integrity values had to be greater than 8 to pass quality control. The majority of samples had values between 8 and 10.

The gene expression data of the first set of 67 BXD RI strains used for system genetics analysis were collected using the Affymetrix Mouse Genome M430 2.0 array based on the manufacturer’s protocol. The gene expression data of the second set of 26 BXD RI strains under saline and ethanol treatment used for analysis of the effect of ethanol on gene expression were collected using the Illumina Sentrix Mouse-6 v1.1 arrays based on the manufacturer’s protocol. Both the first (“Hippocampus Consortium M430v2”) and second data sets (“UTHSC BXD Hippocampus ILM v6.1 NOS Balanced (Feb17) RankInv” and “UTHSC BXD Hippocampus ILM v6.1 NOE Balanced (Feb17) RankInv”) are listed in our GeneNetwork website (www.genenetwork.org). Detailed information for these data sets, including strain, age, sex, experimental protocol, data quality control, etc. can be found in the “info” pages.

### Quantitative RT-PCR

The expression of CD14 gene and several other co-expressed genes were verified using quantitative RT-PCR. We performed quantitative RT-PCR as previously described [27]. Briefly, total RNA was extracted from the hippocampus of 10 BXD strains treated with either ethanol or saline. Three samples were collected from each group for each strain. The total RNA from each individual sample was transcribed into cDNA using a reverse transcription Kit (Invitrogen, Carlsbad,CA) following manufacturer’s instruction. The cDNA was used as the template to amplify the specific products for individual genes using the SYBR Green-based real-time PCR on a LightCycler 4800 real-time PCR instrument (Roche Applied Science; Indianapolis, IN). The relative expression of each gene was normalized to β-actin by using the ΔΔ2Ct method, and data were presented as mean ± SD based on the average of expression levels calculated from all 10 strains by comparing the ethanol group to the saline group. The sequences of the PCR primers are listed in Table 1.

**Table 1:** Primer sequences used for individual genes.

Genes	Primer sequences
CD14	5'CTCTGTCCTTAAAGCGGCTTAC (Forward)
	5'GTTGCGGAGGTTCAAGATGTT (Reverse)
Aif1	5'ATCAACAAGCAATTCCTCGATGA (Forward)
	5'CAGCATTCGCTTCAAGGACATA (Reverse)
CD68	5'TGTCTGATCTTGCTAGGACCG (Forward)
	5'GAGAGTAACGGCCTTTTTGTGA (Reverse)
IL18	5'GACTCTTGCGTCAACTTCAAGG (Forward)
	5'CAGGCTGTCTTTTGTCAACGA (Reverse)
Ly86	5'CTGCCCTCCTTGTGTGGATTC (Forward)
	5'TGGAACACTGGTCAATGGAAAG (Reverse)
βactin	5'GGCTGTATTCCCCTCCATCG (Forward)
	5'CCAGTTGGTAACAATGCCATGT (Reverse)

### Data processing

Raw microarray data collected using the Affymetrix platform were normalized using the Robust Multichip Array (RMA) method [28]. Raw microarray data collected using Illumina platform were normalized using the Rank Invariant method and background subtraction protocols provided by Illumina as part of the BeadStation software suite. Both sets of expression data were then re-normalized using a modified Z score described in a previous publication [29]. We calculated the log base 2 of the normalized values, computed Z scores for each array, multiplied the Z scores by 2, and added an offset of 8 units to each value. This transformation yields a set of Z-like scores for each array that have a mean of 8, a variance of 4, and standard deviation of 2. The advantage of this modified Z score is that a two-fold difference in expression corresponds approximately to a 1 unit change.

### Statistical analysis

Hippocampal gene expression data from saline or alcohol treatment were evaluated using a two-variable (strain, treatment) Analysis of Variance (ANOVA).

### Heritability calculation

The heritability (h^2^) of gene expression was calculated using the broad sense heritability method [30], in which variances among strain means was compared to total variance. The equation used was:

$$h^{2}=\frac{0.5VA}{0.5VA+VE}$$

VA is the variance among strain means and VE is the variance within strains.

### Expression QTL (eQTL) mapping and SNP analysis

Among the BXD strains, differential expression of any given gene can be attributed to polymorphisms (referred to as expression quantitative trait loci or eQTLs) driving that gene’s expression. eQTL mapping was used to identify chromosomal locations associated with differential expression of genes. We performed eQTL analysis using the WebQTL module on GeneNetwork (www.genenetwork.org) according to our published methods [29]. Simple interval mapping was used to identify potential eQTLs regulating *Cd14* expression levels and to estimate the significance at each location using known genotypic data for those sites. Composite interval mapping was also used to control for genetic variance associated with major eQTLs and therefore identify any secondary eQTLs that may have been otherwise masked. Each of these analyses produced a likelihood ratio statistic (LRS) score, providing us with a quantitative measure of confidence of linkage between the observed phenotype—in this case variation in the expression level of *Cd14*—and known genetic markers. The significance of the eQTLs was calculated using more than 2000 permutations tests. Loci were considered statistically significant if genome-wide *p* < 0.05. Sequence variability between B6 and D2 was then determined using the SNP variant browser link on GeneNetwork.

### Gene enrichment analysis

Genetic correlation, partial correlation, and literature correlation were performed to filter a list of transcripts correlated with *Cd14* in order to perform gene enrichment analysis.

Genetic correlation analysis was performed on GeneNetwork to identify transcriptional abundance relationships between *Cd14* and other genes. *Cd14* was compared to all probe sets in the mouse genome. Genes potentially correlated with *Cd14* required an expression level greater than baseline 7.0 as well as a significant correlation with *Cd14*, indicated by the Pearson product correlations value (*p* < 0.05). Genes meeting these criteria were then selected for further analysis.

Partial correlation is the correlation found between two variables that is presents after controlling for and removing the effect of one or more other variables. It can be used to determine the most likely set of *cis*-modulated genes within upstream regulatory regions. In order to find genes significantly correlated with *Cd14*, we performed partial correlation analysis controlling for the genotype at the *Cd14* locus after genetic correlation analysis. Genes that were connected only genetically to the *Cd14* locus were eliminated, leaving genes that were both genetically and biologically related to *Cd14* to be further analyzed.

Literature correlation examines the *r* value for genes that are described by similar terminology in published papers. After genetic and partial correlation, we performed literature correlations using the Semantic Gene Organizer [31] to find the true biological correlation between *Cd14* and other genes. Genes with higher correlation values (*r* > 0.3) were selected for further analysis.

Genes with significant genetic (p < 0.05), partial (*p* < 0.05), and literature correlations (*r* > 0.3) were then selected for gene set enrichment analysis. After removing Riken clones, intergenic sequences, predicted genes, and probes not associated with functional mouse genes, the remaining list of correlates with mean hippocampal expression levels above baseline were uploaded to Webgestalt (http://bioinfo.vanderbilt.edu/webgestalt/) for gene ontology (GO) and pathway analyses [32]. The *p*-values from the hypergeometric test were automatically adjusted to account for multiple comparisons using the Benjamini and Hochberg correction [33]. Categories with an adjusted *p*-value of less than 0.05 indicated that the set of submitted genes was significantly over-represented in those categories.

### Phenotype correlation

To identify phenotypes highly correlated with variation in the *Cd14* gene, we queried the BXD phenotype database in our GeneNetwork website (www.genenetwork.org). Phenotypes, including behaviors related to ethanol, were analyzed for correlations to *Cd14* using Spearman’s product correlations value (*p* < 0.05).

### Genetic network construction

The gene network was constructed and visualized using the Cytoscape utility through “Gene-set Cohesion Analysis Tool (GCAT)” (http://binf1.memphis.edu/gcat/index.py). The nodes in the network represent genes and the edge between two nodes represent cosine scores of Latent Semantic Indexing (LSI) determines if the functional coherence of gene sets is larger than 0.6. The significance of the functional cohesion is evaluated by the observed number of gene relationships above a cosine threshold of 0.6 in the LSI model. The literature *p*-value (LP) is calculated using Fisher’s exact test by comparing the cohesion of the given gene set to a random one [31].

## Results

### *Cd14* expression variance across BXD mice and heritability

**Fig 1:**
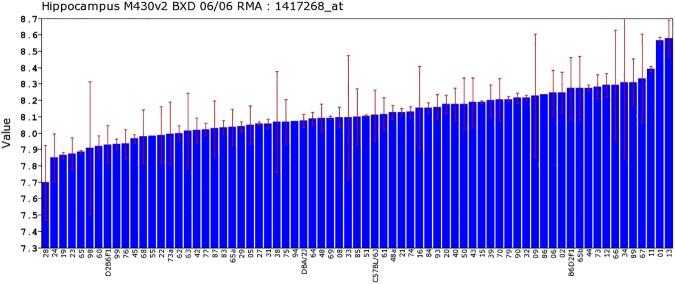
Differential expression of the *Cd14* gene across mouse strains rank ordered by expression levels. The standard deviation and mean expression of *Cd14* in each mouse is shown across the parent DBA/2J and C57BL/6J strains, F1 hybrids, and 67 BXD strains. The x-axis represents the mouse strain, with the y-axis as the mean gene expression using the log2 scale.

The only the probe set representing the *Cd14* gene (1417268_at) targeting the exon and 3’-UTR of the *Cd14* gene in the Affymetrix M430 dataset was used. *Cd14* expression varied widely among 67 BXD RI strains, with a fold-change of 1.84 (Fig 1). The strain with the highest level of expression (8.58±0.11) was BXD13, while the strain with the lowest expression (7.69±0.22) was BXD28. The heritability of *Cd14* expression value was 0.323, which suggested that genetic factors contribute to variation in expression. This heritable variation enables us to identify genetic loci that influence expression of *Cd14* in the BXD mice.

### eQTL mapping and sequence variants of *Cd14*

**Fig 2:**
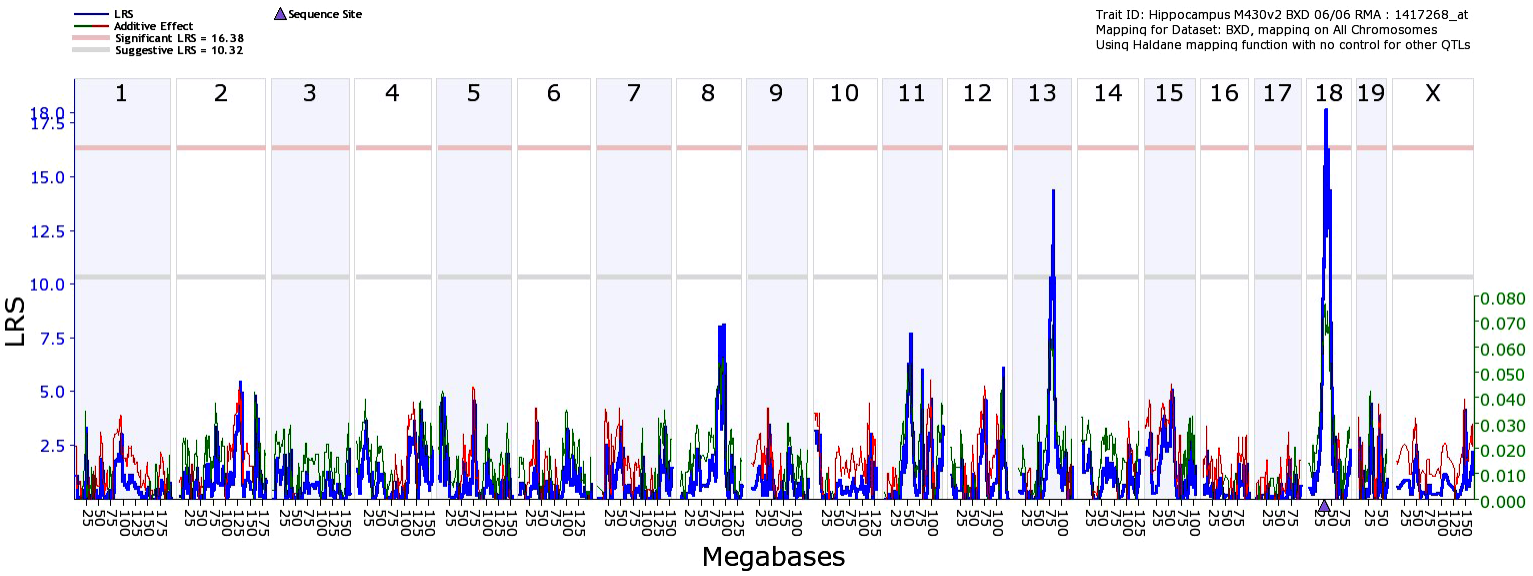
Interval mapping for *Cd14* was done using the Affymetrix dataset to identify significant eQTLs in the mouse genome. The x-axis denotes a position on the mouse genome, in megabases (Mb), while the y-axis gives the likelihood ratio statistic scores (LRS), a measurement of the linkage between differences in *Cd14* and differences in the region of the genome. The blue lines indicate the LRS values at a given position, with a significant LRS at 16.56 and suggestive LRS at 10.39. There was a significant eQTL on chromosome 18, around 36–37 Mb, and a suggestive eQTL on chromosome 13.

The Affymetrix M430 database was used to identify sequence variants affecting the expression of *Cd14* via eQTL mapping. The gene that codes for *Cd14* is located at 36.88 Mb on chromosome 18. Simple interval mapping found a significant eQTL with a likelihood ratio statistics (LRS) of 18.2 on chromosome 18 at the *Cd14* gene location (Fig 2). Composite interval mapping revealed no secondary loci modulating *Cd14* expression levels. This indicates that *Cd14* is *cis*-regulated, meaning that a sequence variant affecting its expression is located within or near the *Cd14* gene itself. Using new open access sequence data resources at GeneNetwork, we identified 3 SNPs in *Cd14* between the BXD parental strains. All three SNPs are located in the coding region, one of which is a synonymous SNP, and the other two nonsynonymous SNPs, indicating that there has been a change in the coded protein (Table 2). At least one of these SNPs is responsible for *Cd14* expression differences in BXD mice.

**Table 2:** The single nucleotide polymorphisms (SNPs) of the *Cd14* gene.

SNP ID	Chr	Mb	Alleles	Gene	Exon	Function	B6	D2
wt37-18-36885521	18	36.885521	C/T	Cd14	2	Synonymous	C	T
rs8255713	18	36.885972	T/A	Cd14	2	Nonsynonymous	T	A
rs8255712	18	36.885973	C/G	Cd14	2	Nonsynonymous	C	G

### Expression differences of *Cd14* between saline control and ethanol groups

We used the Illumina NOS and NOE datasets to analyze the effect of alcohol on the expression of hippocampal *Cd14* gene in BXD mice using a two-way ANOVA with treatment and strain as the between subjects factors. The average of *Cd14* expression across BXD strains in the Illumina NOS group was 7.378, while the average for the Illumina NOE group was 7.318, indicating a decrease in expression after ethanol treatment. Statistical analysis also showed a significant effect of treatment and strain on *Cd14* transcript abundance (Table 3).

**Table 3:** Two-way ANOVA analysis of CD14 expression.

Source	df	Sums of Squares	Mean Square	F-ratio	Prob
Const	1	2807.8	2807.8	815079	0.0001
treatment	1	0.046067	0.046067	13.373	0.0012
strain	25	0.42753	0.017101	4.9643	0.0001
Error	25	0.086121	0.003445		
Total	51	0.559717			

### Gene function enrichment

The expression of 5218 transcripts in the hippocampus was significantly correlated with that of *Cd14* (*p* < 0.05) in the Affymetrix dataset. However, only 524 known transcripts were left after filtering by genetic, partial, and literature correlations (S1A Table). Among these genes, 79 (or about 15% of 524 genes) had significant expression changes after ethanol treatment (S1B Table). These two sets of genes, the collection of 524 genes and the ethanol treated gene set of 79 genes, were submitted for gene ontology analysis to identify those categories with over-represented biological and molecular functions. For the whole set of genes, significant categories for biological processes included: “immune system process” (126 genes, adjP≤2.58e-48), “cell death” (106 genes, adjP≤1.95e-28), “response to chemical stimulus (136 genes, adjP≤3.62e-35), and “response to stress” (136 genes, adjP≤5.38e-34). The significant categories for molecular function were: “cytokine binding” (9 genes, adjP≤6.22e-05) and “cytokine receptor binding” (20 genes, adjP≤2.59e-07). For the gene set with significant expression change after ethanol-treatment, significant categories for biological processes included: “immune system processes” (20 genes, adjP≤-2.97e-07), “response to chemical stimulus” (26 genes, adjP≤5.49e-08), and “inflammatory response” (9 genes, adjP≤8.83e-05). A significant category for molecular function was “lipopolysaccharide binding” (2 genes, adjP≤1.07e-02). The gene ontology result for the whole data set is listed in S2 Table.

The WikiGenes (https://www.wikigenes.org/) database was used to identify pathways in which these genes are involved. Gene pathway analysis for the whole gene set resulted in 75 significant pathways (adj P<0.05, S3 Table). Among the top 20 pathways, most are involved in immune system function, including multiple IL-Signaling Pathways, B Cell Receptor Signaling Pathway, T Cell Receptor Signaling Pathway, the chemokine signaling pathway, MAPK signaling pathway, toll-like receptor signaling pathways, etc. (Table 4). Pathway analysis for the ethanol treated genes resulted in 3 significant pathways: macrophage markers, MAPK signaling pathway, and G protein signaling pathways, all of which are among the top 20 pathways from the whole gene set.

**Table 4:** Top 20 *Cd14* gene pathways.

PathwayName	Number of Genes	Raw P value	Adjusted P value
MAPK signaling pathway	25	2.57E-15	2.80E-13
Focal Adhesion	23	3.56E-12	1.94E-10
Chemokine signaling pathway	21	4.75E-11	1.29E-09
Toll Like Receptor signaling	17	3.69E-11	1.29E-09
IL-6 signaling Pathway	16	1.93E-09	4.21E-08
MicroRNAs in cardiomyocyte hypertrophy	15	2.79E-09	5.07E-08
B Cell Receptor Signaling Pathway	20	3.97E-09	5.80E-08
Insulin Signaling	18	4.26E-09	5.80E-08
Apoptosis	14	5.10E-09	6.18E-08
IL-2 Signaling Pathway	14	5.91E-09	6.44E-08
IL-3 Signaling Pathway	15	7.19E-09	7.12E-08
IL-4 signaling Pathway	12	3.97E-08	3.61E-07
T Cell Receptor Signaling Pathway	15	1.67E-07	1.40E-06
IL-7 Signaling Pathway	9	2.55E-07	1.99E-06
Integrin-mediated cell adhesion	12	5.93E-07	4.31E-06
IL-5 Signaling Pathway	11	7.23E-07	4.93E-06
Alzheimers Disease	10	2.08E-06	1.33E-05
EGFR1 Signaling Pathway	17	2.76E-06	1.67E-05
Toll-like receptor signaling pathway	7	8.10E-06	4.65E-05
GPCRs, Class A Rhodopsin-like	14	9.19E-06	5.00E-05

### Gene network

**Fig 3:**
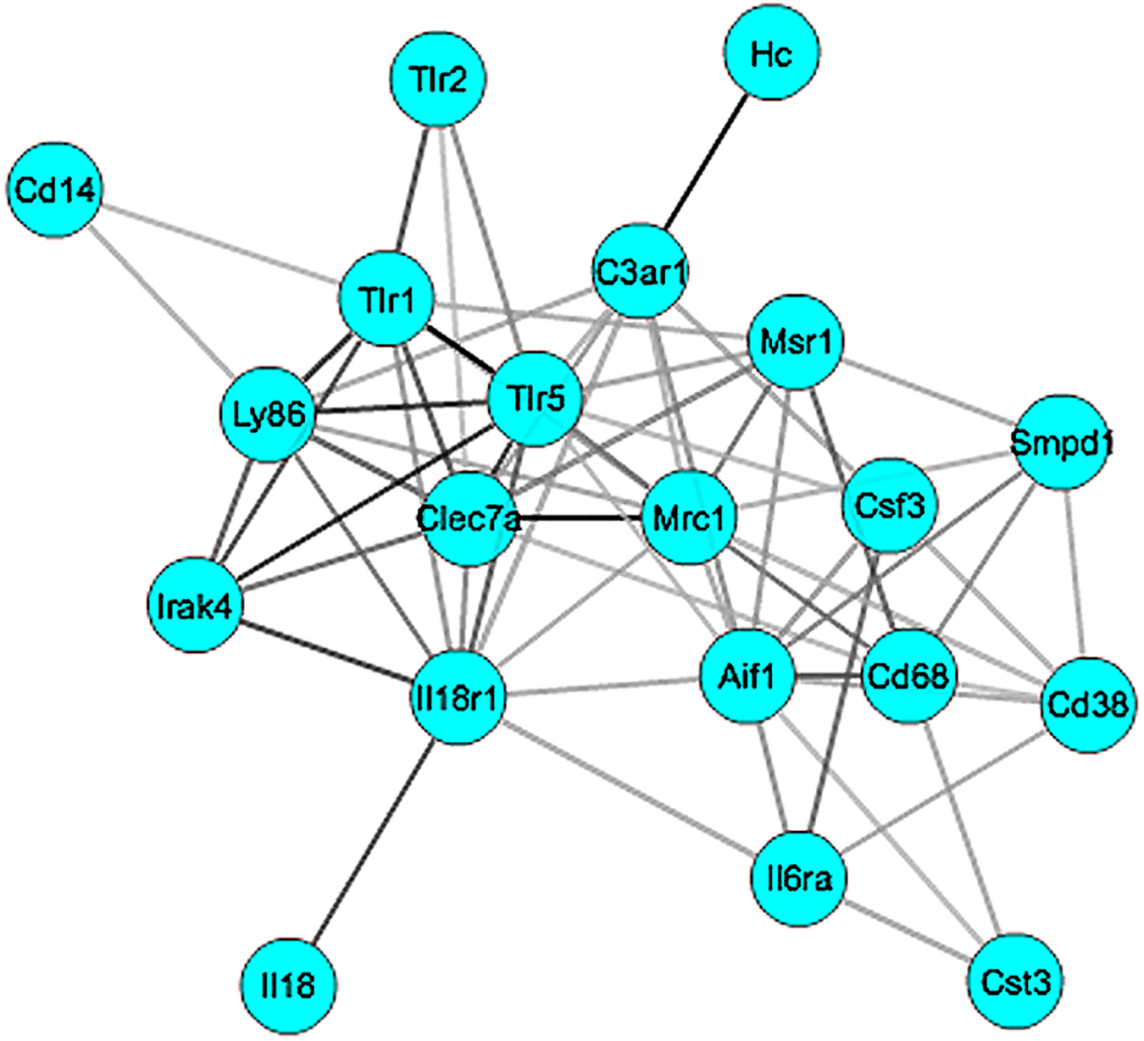
The gene network of *Cd14*. The 20 genes showed significant functional cohesion with a literature p value of8.10365e-17. Each node represents a gene, and each line between genes represents the cosine score of Latent Semantic Indexing (LSI), which is the functional coherence of gene sets.

The top 20 transcripts of the whole gene set were uploaded to GCAT (http://binf1.memphis.edu/gcat/index.py) for functional coherence analysis and gene network construction. These genes showed significant functional cohesion with a literature *p* value of < 8.10365e-17 (Fig 3). Multiple resources, including Chillibot, GeneCard, and Pubmed, were used to determine whether members of the *Cd14* co-expression network had been previously associated with ethanol. Of the genes in the identified network, *Tlr2*, *Il18*, *Cst3*, *Csf3*, and *Smpd1* were significantly correlated with ethanol-related phenotypes reported in the literature. *Cd68*, *Il18*, *Ly86*, *Aif1*, and *Cd14*all evinced significant changes in expression after ethanol treatment in our experiment. Overall, around 50% of the genes identified in the gene network have either experimental or literature support that they are related to both *Cd14* and have differential expression after ethanol treatment.

### Quantitative RT-PCR validation

**Fig 4:**
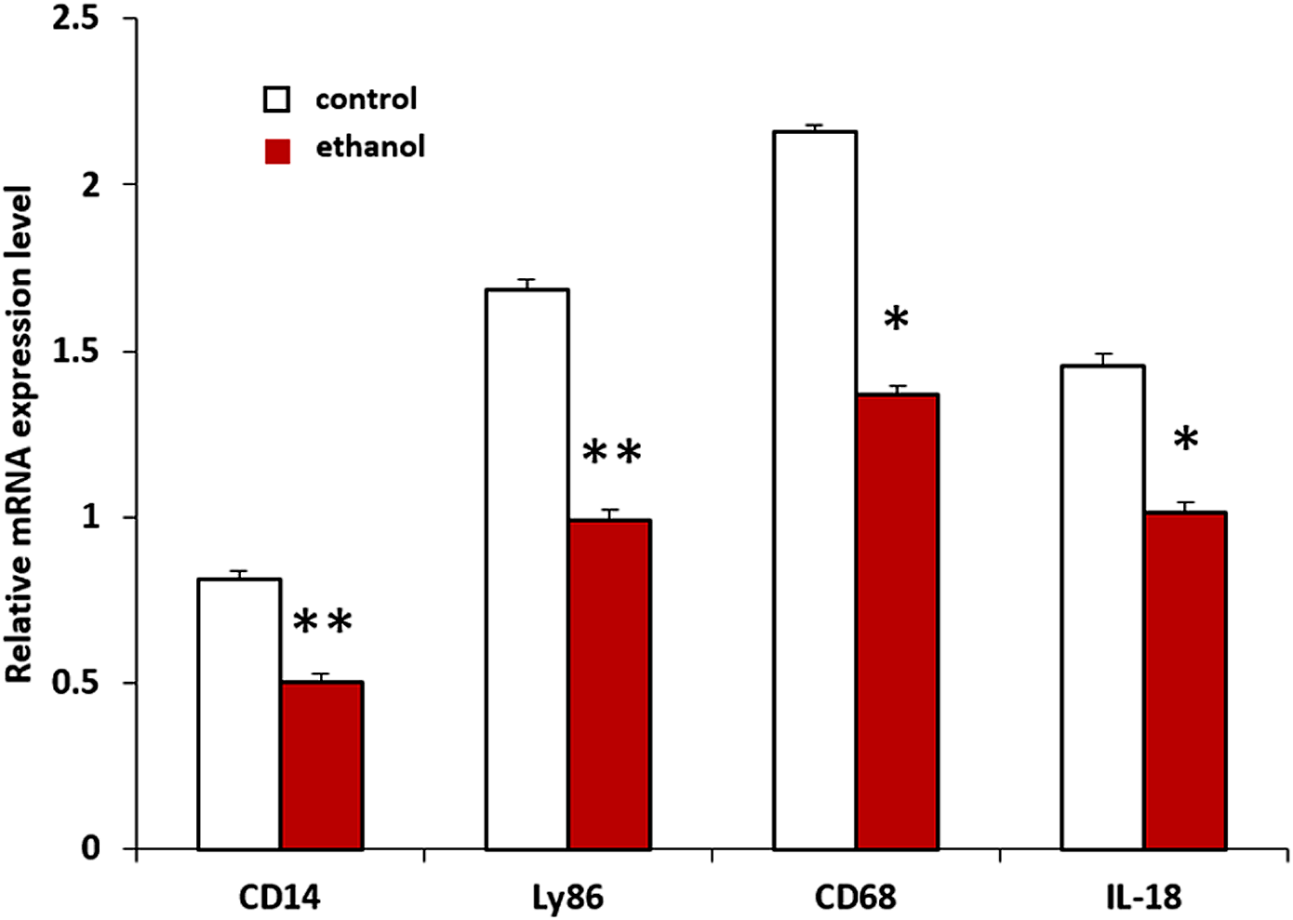
Gene expressions for *Cd14*, *Ly86*, *Cd68* and *Il18* in the ethanol and saline treated hippocampus were detected by quantitative RT-PCR. Data were presented in relative expression level averaged from all 10 strains in ethanol and saline treated mice (*p<0.05, **p<0.01).

Genes in the *Cd14* gene network that we found to have significant expression changes after ethanol treatment in our microarray experiment were selected for RT-PCR analyses to verify their expression in the hippocampus across all 10 strains. The RT-PCR result showed that expressions of *Cd14*, *Ly86*, *CD68* and *Il18* were significantly decreased after ethanol treatment compared with the saline group (Fig 4), which was consistent with the results from the microarrays. However, the expression of *Aif1* was very low, and a difference between the two groups could not be detected. The expression of *Cd14* was decreased by approximately 40% in ethanol group when compared with the saline group (F = 9.6471, P<0.0064). The expression of *Ly86*, *Cd68*, and *Il18* were decreased by 42% (F = 3.3889, P<0.0027), 37% (F = 8.0179, P<0.0114), and 31% (F = 6.7052, P<0.0191) respectively in the ethanol group compared to the control group.

### Phenotypic correlations

To show the extent to which *Cd14* is directly associated with ethanol-related phenotypes, we performed correlational analyses with phenotypes archived in our GeneNetwork database, and identified 18 ethanol-related phenotypes significantly correlated with *Cd14* expression, including ethanol consumption, neurodegeneration, and degree of locomotor stimulation following exposure to alcohol (Table 5).

**Table 5:** The phenotypes that are significantly correlated with alcohol.

Record ID	Phenotype	N	P value	Authors
18669	Cell death in forebrain following exposure to ethanol at day 9 of gestation (E9), TUNEL-positive apoptotic cells (subtracted control maltose-dextranes counts) [cells/mm2]).	18	0.018	Balce, K.
12635	Anxiety assay (E5_NR), restraint stress (15 min) + ethanol treated (1.8 g/kg i.p.), number of rears in the light side using a light-dark box in 8–12 week-old males during 5 min session [n beam breaks].	26	0.033	Putman AH, and colleagues
10988	Anxiety assay after restraint stress (15 min) and an ethanol injection (1.8 g/kg ip), rearing events in the light side using a light-dark box by 8–12 week-old males during a 10 min session [n]	26	0.036	Putman AH, Miles MF
10066	Ethanol response (1.5 g/kg), blood ethanol concentration (BEC) normalization data set (BEC was stabilized to ~1.5 mg EtOH/ml blood using daily pyrazole injections) [mg/ml]	23	0.003	Crabbe JC
10349	Ethanol response (1.75 mg/kg ip), time to ataxia measured as loss of balance using a dowel test (Loss corresponds to BEC time 0) [min]	27	0.026	Kirstein SL, Davidson KL, Ehringer MA, Sikela JM, Erwin VG, Tabakoff B
10089	Ethanol response (2 g/kg ip), locomotor tolerance or sensitization in males, difference between activity after fourth ethanol trial and first trial [cm]	19	0.025	Cunningham CL
11717	Ethanol response (2.25 g/kg ip), motor coordination effects, difference in time on rotarod between training session and ethanol for females [sec]	55	0.048	Philip VM, Ansah TA, Blaha CD, Cook MN, Hamre KM, Lariviere WR, Matthews DB, Mittleman G, Goldowitz D, Chesler EJ
18114	Ethanol response (20% v/v) using the drinking in the dark (DID) method (4 hr access on day 4 of DID) to measure average baseline drinking under 5 weeks of normal housing [g/kg]	15	0.035	Jones BC, Lu L, Mormede P, Mulligan MK, Terenina E, Williams RW, Zhao W
13566	Ethanol response (20% v/v), consumption using drinking in the dark (DID) method (4 hr access on day 4 of DID) in females, baseline in chronic mild (CMS) stress group 1 week before start of 7 weeks of CMS (Ph	13	0.017	Jones BC, Lu Lu, Williams RW
18108	Ethanol response (20% v/v), consumption using drinking in the dark (DID) method (4 hr access on day 4 of DID) in females, difference between average alcohol consumption during first 5 weeks and last 4 weeks	14	0.046	Jones BC, Lu L, Mormede P, Mulligan MK, Terenina E, Williams RW, Zhao W
15961	Cell death in forebrain following exposure to ethanol at E9 (TUNEL+ cells/mm2)	14	0.007	Boyle, J. & Goldowitz, D.
12970	Ethanol response (chronic intermittent ethanol CIE cycles), 3a,5a-tetrahydroprogesterone (THP, allopregnanolone) in plasma 72 h after the 5th cycle of ethanol vapor chamber treatment, 16 to 18 week-old males	14	0.010	Morrow AL, Lopez MF, Becker HC, Miles MF, Williams RW
13022	Ethanol response (CIE), 3a,5a-androsterone in blood plasma 3 days after cycle 5 of chronic intermittent ethanol vapor [pg/g]	18	0.018	Morrow, Lopez, Becker, Miles and Williams
13019	Ethanol response (CIE), 3a,5b-THP in blood plasma 3 days after cycle 5 of chronic intermittent ethanol (CIE) vapor treatment [pg/g]	19	0.004	Morrow, Lopez, Becker, Miles and Williams
13027	Ethanol response control, 3a,5a-tetrahydrodeoxycorticosterone (THDOC) in blood plasma 3 days after cycle 5 of chronic intermittent air vapor (CIE air control) [pg/g]	15	0.032	Morrow, Lopez, Becker, Miles and Williams
13561	Ethanol hepatocyte cytotoxicity response 9 hr after treatment (2.0 M), ratio of ethanol-induced release to maximal (lysis) lactate dehydrogenase (LDH) release, young adult male liver	21	0.032	Kaiser R, Hoynowski S, Williams RW, Scott RE
16236	Hepatocyte damage in vitro measured as difference in lactate dehydrogenase (LDH) release relative to untreated hepatocytes, after 48 hrs 5 ng/ml TGF-beta + 0.6% ethanol, median in males [% LDH increase]	21	0.045	Liebe R, Hall RA, Williams RW, Dooley S, Lammert F
17399	Ethanol-induced conditioned taste aversion, saccharin intake during trials 2–4 after 4 g/kg ethanol injections (Group 4 g/kg) in 60 to 130-day-old males (from Table 1, upper right pa	19	0.026	Risinger FO, Cunningham CL

## Discussion

In this study, we aimed to elucidate the relationship between ethanol treatment and the expression of *Cd14* in the hippocampus as well as to identify interacting genes and pathways through which *Cd14* regulates ethanol responses. We found that *Cd14* shows variable expression in the hippocampus among the BXDs and is *cis*-regulated in the Affymetrix hippocampus dataset, which makes this gene an excellent candidate for study as a modifier that regulates expression of other transcripts and biologic phenotypes [34]. *Cd14* was not found to be *cis-*regulated in the Illumina hippocampus datasets that were used for this paper, but it was found to be *cis*-regulated in the Illumina hippocampus combined dataset that has more samples per strain and increased power, allowing the *cis*-eQTL to be detected (S1A Fig). To verify that *Cd14* is *cis-*regulated, we also checked all available BXD datasets in GeneNetwork and found that *Cd14* is also a *cis*-regulated in the neocortex, mid-brain, prefrontal cortex, striatum, ventral tegmental area, bone, kidney, progenitor cells, and spleen among BXD mice (S1 Fig), which were generated using different microarray platforms. This data strongly supports that *Cd14* is *cis-*regulated and suggest that a polymorphism in *Cd14* between B6 and D2 mice affect the expression of *Cd14* among BXD mice. Binding of a transcription factor to the promoter region of a gene is traditionally regarded as the main mechanism by which gene expression is regulated. Recent studies have shown, however, that polymorphisms within both intronic and exonic regions also play an important role in regulating gene expression [35, 36]. In our study we found three SNPs in the exonic regions of the *Cd14* gene. At least one of these SNPs is responsible for *Cd14* expression differences in multiple tissues among BXD mice.

Phenotypic correlations showed us broadly that *Cd14* expression and ethanol responses/effects are indeed related; we identified a number of such ethanol-related phenotypes. These included ethanol consumption, ethanol conditioned taste aversion, ethanol-induced locomotion, ethanol-induced anxiety, and embryonic neurocellular death. These associations, however, still do not provide insight into the mechanisms by which ethanol and *Cd14* interact.

Although the information provided is a blunt instrument, GO analyses do provide some insight into the possible genetic, biological and molecular intersections of *Cd14* and ethanol. For example, “response to chemical stimulus” and “response to stress” were functions that were found when we examined the whole gene set and the ethanol-related gene set. GO analysis found that cytokine and cytokine receptor binding is a vital part of *Cd14* and its correlated genes’ processes.

In addition to regulating proinflammatory cascades and LPS binding, *Cd14* also plays a role in monocyte apoptosis. GO analysis identified “cell death,” along with “apoptotic process” and “programmed cell death,” as significant biological categories. Apoptosis and programmed cell death are innate immune system functions; CD14 also recognizes apoptotic cells [37]. Increased *Cd14* expression is associated with monocyte survival whereas decreased *Cd14* expression is associated with monocyte apoptosis [38]. There are also numerous reports on ethanol-mediated cell death. For example, a study has shown that chronic alcohol consumption activates glial cells in rats, which upregulates inflammatory mediators. This process occurs along with the stimulation of IRAK and MAP kinases, which in turn, activate NF-κB and AP-1, which are associated with apoptosis and inflammatory damage [39].

Overall, it is unclear whether alcohol directly affects *Cd14* expression and through which mechanisms, but our findings present potential biological processes that may be involved. Although we identified proinflammatory cascades, LPS binding, and apoptosis as potential mechanisms through which *Cd14* regulates ethanol response, the majority of the genes we identified as potential players in the *Cd14* network have immune-related functions.

We used the top 20 genes correlated with *Cd14* expression to create a *Cd14* gene network. Several genes in the *Cd14* gene network showed significant changes in expression after ethanol treatment in our experiment. These genes included *Aif1*, *Cd68*, *Il18*, *Ly86*, and *Cd14*. We further verified these changes using RT-PCR after analysis of our data. Four out of these 5 genes (*Cd68*, *Il18*, *Ly86*, and Cd14) have been confirmed to have significant changes in expression after ethanol treatment. Among these genes with ethanol-related changes in gene expression, only *IL18* was significantly correlated with ethanol based on previous literature. We present these genes as potential players in the *Cd14* gene network and its involvement in regulating ethanol’s effects on the immune system. Interleukin 18 (*Il18*) is a proinflammatory cytokine involved in cell immunity. IL18 responds to LPS, and its secretion is CD14-dependent [40], but ethanol has been found to reduce LPS-stimulated secretion of IL18 [41].

Cluster of differentiation 68 (CD68), similar to CD14, is present on macrophages and is often used as a marker of macrophage activation. The effects of ethanol on macrophage activation are well described [42]. CD68 mRNA levels have been shown to be positively correlated with inflammation of adipose tissue [43]. Ethanol may attenuate this inflammation through its ability to decrease Cd68 mRNA, which is associated with lower levels of adipokines [44].

Methylation of the *Ly86* gene has been associated with obesity, insulin resistance, and inflammatory markers [45]. Although there are only a few studies on the *Ly86* gene, given ethanol’s effects on inflammation, it is possible that ethanol could affect expression of the *Ly86* gene as well.

Among the 20 genes identified in the network, five of them (*Tlr2*, *Il18*, *Cst3*, *Csf3*, and *Smpd1*) are significantly correlated with alcohol exposure based on literature reports. Thus, they may interact with *Cd14* to regulate ethanol responses.

Recall that CD14 is a co-receptor of TLR-4 and that the toll-like receptors are critical to innate immune responses. Here we identified TLR-2 as one of the genes that was found to be significantly correlated with ethanol exposure through literature reports. The toll-like receptor 2 (TLR2, also known as CD282) is a membrane protein expressed on microglia, monocytes, macrophages, B-, C- and T-cells that aids in recognizing foreign substances and inducing a proinflammatory cytokine cascade [46]. It is downregulated through inhibition of p38 and ERK1/2 pathway activation, which is also involved in the CD14/TLR4 pathway [47]. Ethanol-treated mice and human alcoholics have increased expression levels of TLR-2, TLR-3, and TLR-4 in the orbital frontal cortex [48].

Cystatin C (*Cst3*), a marker gene for kidney function, was identified in our network *Cst3* is primarily a marker of kidney function, although it is found in many organs. It is a cysteine protease inhibitor, but has protective functions as well, as decreased expression has been associated with neurodegenerative diseases [49]. Like many other genes identified in our study, it is also linked to inflammation [50]. *Cst3* has previously been found to be upregulated in the midbrain after ethanol exposure [51].

The remaining genes have a variety of functions. Granulocyte-colony stimulating factor 3 (*Csf3*) stimulates the release granulocytes and stem cells from bone marrow into the bloodstream. Ethanol has been shown to downregulate *Csf3* [52]. *Smpd1*, or sphingomyelin phosphodiesterase, codes for acid sphingomyelinase, which is a lipid hydrolase. Mutations in the *Smpd1* gene cause types A and B Niemann-Pick disease, which is a family of metabolic disorders. While *Smpd1* mRNA levels have been shown to increase with chronic alcoholic liver disease and following alcohol exposure [53], other studies have found no changes in *Smpd1* mRNA levels in alcohol-fed mice [54, 55].

Together, the aforementioned genes we have identified as potential members of the *Cd14* network and their functions are supported by the GO categories we also identified, including “response to chemical stimulus,” “immune response,” and “inflammatory response.” Many of these genes have connections to pathways that are correlated with *Cd14*, such as the MAPK p38 signaling and chemokine signaling pathways. The p38 MAPK pathway is essential in the synthesis of proinflammatory cytokines, and p38 MAPK may regulate inflammatory gene expression on the post-transcriptional level [56]. Toll-like receptor pathways are involved in apoptosis, inflammatory responses and T-cell receptor (TCR) stimulation; TLR4, specifically, works along with CD14 to recognize lipopolysaccharides [57]. Activation of TCR results in the synthesis of cytokines, similar to CD14’s ability to induce the NF-κB cascade to produce cytokines [58]. Transforming growth factor beta, or TGF-β is a cytokine secreted by macrophages that likely inhibits the release of cytokines by inhibiting translation of TNF-α mRNA [59]. Adipogenesis, which is the process in which preadipocytes differentiate into adipocytes, may also involve *Cd14*. Acute inflammation of adipose tissue is important in tissue protection, remodeling, and expansion [60], and *Cd14* modulates adipose tissue inflammatory activity [61]. Most of the evidence suggest that the cytokine signaling pathway and proinflammatory cascade represent a significant intersection for these pathways.

Ethanol has a significant effect on several signaling pathways. The proinflammatory cytokine and chemokine cascades are attenuated by drinking through a variety of pathways, including the MAPK pathway [42]. Ethanol also downregulates p38 MAPK levels [62]. Chemokine receptors, which detect chemokines that are formed through proinflammatory stimuli such as TNFα, are G protein coupled receptors (GPCR). GPCRs are known to be implicated in neurodegeneration, Alzheimer’s disease, and inflammation, all of which are affected by alcohol consumption [63]. Ethanol can also attenuate immune responses through increased macrophage and monocyte TGF-β levels, thus lowering proinflammatory cytokine levels [64] and promoting macrophage apoptosis [65]. There is strong evidence that ethanol affects these pathways through inflammation or the immune response.

This study presents these pathways as potential paths through which *Cd14* and ethanol interact and demonstrates that they are involved in the regulation of ethanol in the immune system.

## Conclusion

The interaction between ethanol and the immune system is complicated and intricate, with many pathways and genes acting and interacting to regulate ethanol response. We found significant expression changes in the *Cd14* gene, which is an essential part of the proinflammatory cytokine cascade and expressed primarily on macrophages. To further study this change, we identified GO categories for a gene set with significant correlations to *Cd14* and a gene set with significant correlations to ethanol and *Cd14*. We also identified several pathways that may mediate ethanol’s response through *Cd14* and constructed a possible gene network for *Cd14*. In this study, we present these pathways and gene network as part of ethanol responses in the immune system through *Cd14*. Further investigation is needed to elucidate the nature of these relationships. While *Cd14* is recognized as a co-receptor of toll-like receptors, many of its other functions/roles are yet to be characterized. Our identification of genes in the *Cd14* gene network and pathways may be a step toward identifying additional roles of *Cd14*.

## Supporting information

S1 Table A. Genes with high expression that are significantly correlated with *Cd14*.
B. Genes with high expression after ethanol treatment that are significantly correlated with *Cd14*.
(XLSX)

S2 Table Complete list of gene ontology categories associated with *Cd14* and its correlated genes. (XLSX)

S3 Table Complete list of pathways associated with *Cd14* and its correlated genes. (XLSX)

S1 Fig Interval mapping of Cd14 in various GeneNetwork BXD datasets in which Cd14 is a cis-regulated gene. (DOCX)
