# Supplementary material for: The effect of alcohol on the differential expression of cluster of differentiation 14 gene, associated pathways, and genetic network
Source: PLoS One. 2017 Jun 2;12(6):e0178689. doi: 10.1371/journal.pone.0178689 (PMC5456352; doi:10.1371/journal.pone.0178689)
Supplement: S1 Fig — (DOCX) [file pone.0178689.s004.docx]

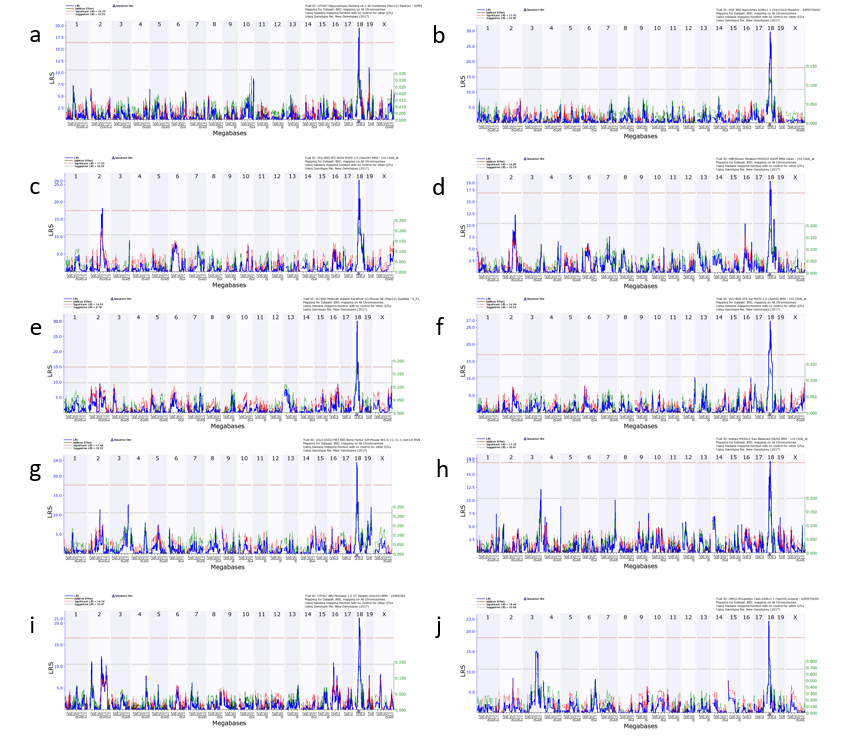


**Supplement Figure 1. Interval mapping of *Cd14* in various GeneNetwork BXD datasets in which *Cd14* is a *cis-*regulated gene.** *Cd14* is a *cis-*regulated gene in various tissues, whose datasets can be found on GeneNetwork. The x-axis represents a position on the mouse genome, in megabases (Mb), while the y-axis gives the likelihood ratio statistic scores (LRS). The blue lines indicate the LRS values at a given position, with a significant LRS at 16.56 and suggestive LRS at 10.39. There is a significant eQTL on chromosome 18 for the a) hippocampus in “UTHSC Hippocampus Illumina v6.1 All Combined (Nov12),” b) neocortex in “HQF BXD Neocortex ILM6v1.1 (Dec10v2) RankInv,” c) prefrontal cortex in “VCU BXD PFC EtOH M430 2.0 (Dec06) RMA,” d) striatum in “HBP/Rosen Striatum M430V2 04/05 RMA Clean,” e) midbrain in “VU BXD Midbrain Agilent SurePrint G3 Mouse GE (May12) Quantile,” f) ventral tegmental area in “VCU BXD VTA Sal M430 2.0 (Jun09) RMA,” g) femur bone in “UCLA GSE27483 BXD Bone Femur ILM Mouse WG-6 v1. V1.1 (Jan13) RSN,” h) kidney in “Kidney M430v2 Sex Balanced 08/06 RMA,” i) spleen in UTHSC Affy MoGene 1.0 ST Spleen (Dec10) RMA,” and j) progenitor cells in “UMCG Progeitor Cells ILM6v1.1 (Apr09) original.”
